# The Possibility of New Complex Magnet Materials

**DOI:** 10.1002/adma.202518751

**Published:** 2026-03-25

**Authors:** G. Jeffrey Snyder

**Affiliations:** ^1^ Department of Materials Science and Engineering Northwestern University Evanston IL USA; ^2^ Advanced Research Projects Agency ‐ Energy ARPA‐E Washington D.C. USA

**Keywords:** ferromagnet, high‐throughput, materials discovery, motor

## Abstract

Magnets are essential for mobile consumer electronics, electric motors that power industry and the future of transportation as well as generating and transforming most electric power. Strong magnets reduce the size and weight of motors and generators as well as improve efficiency. The most powerful ${\mathrm{Nd}}_{2}\mathrm{Fe}$ 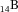
‐based magnets have a complex structure like millions that are expected to exist but have not been made and characterized. With the recent developments of AI materials discovery techniques, which enable data‐driven and machine‐learning‐assisted screening, together with computational approaches that can accurately predict intrinsic magnetic properties of a given structure, and high‐throughput autonomous labs, the discovery of new, ultra‐powerful magnet materials with saturation magnetization greater than 2.5 Tesla or magnetic energy density ($BH_{max}$) greater than 800 kJ/$m^{3}$ is now quite possible.

## Graphical abstract

The quest for stronger magnets enters a new era. With the developments of AI‐driven materials discovery, computational prediction of magnetic properties, and autonomous laboratories, researchers can now explore millions of untested structures. Discovery of next‐generation ultra‐powerful magnets – critical for electric vehicles, renewable energy, and electronics – exceeding 2.5 Tesla magnetization or 800 kJ/$m^{3}$ energy density is now within reach.

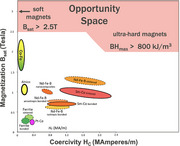

## 1 Introduction

Magnets are ubiquitous in everyday items, providing essential electronic, mechanical and communications functions in mobile consumer electronics, robotics and automobiles [1, 2, 3, 4, 5, 6]. They are especially important for energy applications, necessary for power generation, transformation, and use. Almost all electricity is produced by electric generators using magnets with a large fraction consumed by magnetically driven motors. With the expected electrification of transportation [7] most movement will eventually be powered by magnets.

Magnets in an electric generator use mechanical energy to push electrons, much like a riverboat paddle wheel (Box 1) pushes water upstream, converting the mechanical energy of the spinning generator into the potential energy (voltage) of electrons. At the other end of the power line, another magnet in an electric motor converts the energy of flowing electrons back into mechanical energy. A more powerful magnet is like a bigger paddle that can make motors and generators smaller and lighter. This interconversion between mechanical and electrical energy enables power plants to drive factory motors hundreds of kilometers away, where the losses are primarily from the transmission lines. With roughly 40 percent of all electricity produced used to drive motors, even a small improvement in efficiency has the potential for a large impact.

Most large, stationary generators and motors use electromagnets. These use large coils of copper wire and require electric power to create the magnetic field. Electromagnets are assisted by soft magnet material. A magnet material is ferromagnetic meaning it creates a spontaneous magnetic field at the microscopic scale. Motors, generators, transformers, inductors use soft magnets that switch their magnetic direction with alternating current in an electromagnet.

The maximum magnetic field produced by a ferromagnet is determined by the saturation magnetization $B_{s}$, related to the density of unpaired electron spins. Most magnets use iron‐based alloys or compounds that have at most $B_{s}=2.2$ T, or about two unpaired spins per atom. Given that there are many elements or ions with five or more unpaired spins, higher $B_{s}$ should be possible. New soft ferromagnets [8] with $B_{s}>2.5$ T would find many applications in high‐performance motors, transformers, and inductors, increasing power density for power conversion and electronics.

**FIGURE 1:**
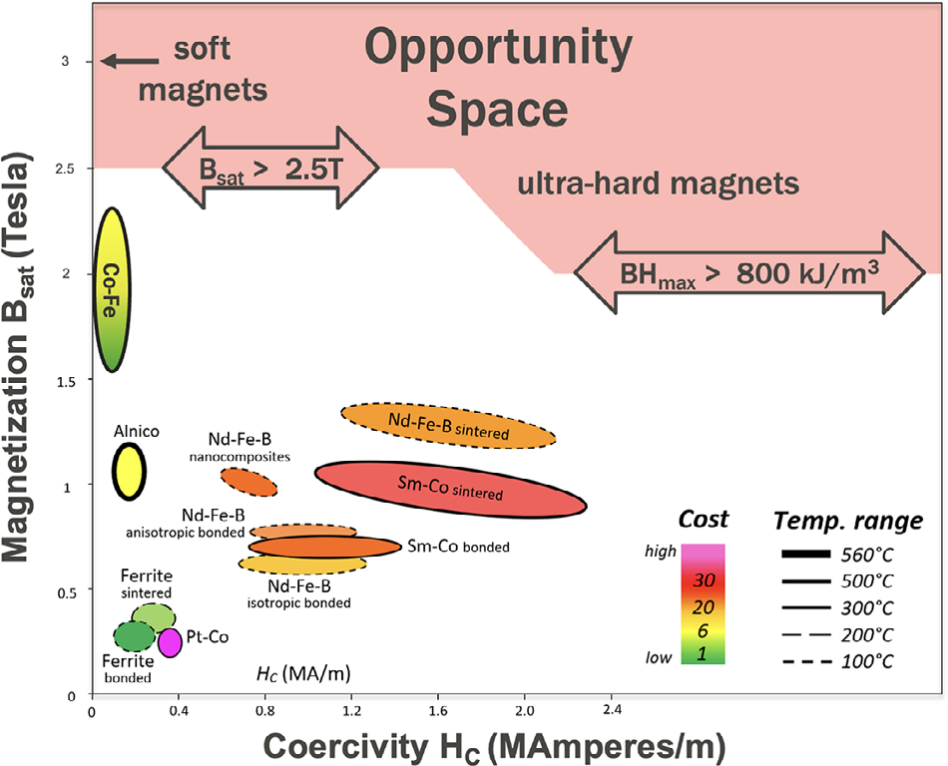
Opportunity space for ultra‐strong magnets with either saturation magnetization $B_{s}>2.5\;T$ or $BH_{\max}>800\;\mathrm{kJ}\;m^{-3}$ at room temperature. Adapted under the terms of the CC BY‐SA 4.0 license [12]. S. Zurek, E‐Magnetica.pl.

**FIGURE 2:**
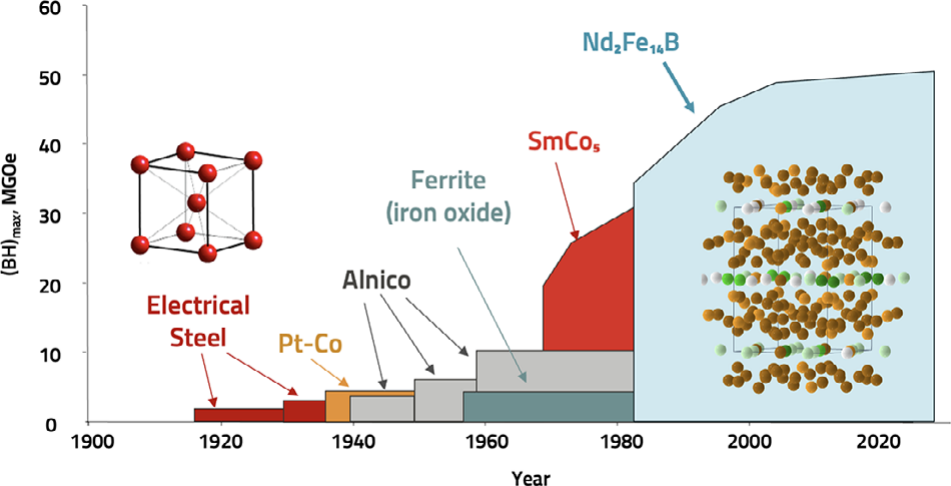
Progression of $BH_{\max}$ over the past century. Until the discovery of ${\mathrm{Nd}}_{2}\mathrm{Fe}$ 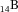 with its complex crystal structure, the best commercial magnets had relatively simple crystal structures. No stronger magnet has been discovered since. Adapted under the terms of the CC‐BY license [4]. Copyright 2012, Springer Nature.

The size and weight of motors and generators can be greatly reduced by replacing half of the electromagnets with permanent magnets. Permanent, or hard, magnets are ferromagnetic materials that provide a strong magnetic field without the need for copper coils or electrical power, making motors and generators smaller and more efficient. For example, permanent magnets enable larger, taller wind turbines that create more power at lower cost [2, 9]. Similarly, strong magnets allow smaller, lighter, and more efficient motors for higher‐performance electric vehicles, and are an enabling technology for drones and electric aviation [7, 10]. Permanent magnets have a high coercivity, $H_{c}$, as well as high magnetization, $B_{s}$, where the best metric is the maximum B×H product, $BH_{max}$. Since the serendipitous discovery of ${\mathrm{Nd}}_{2}\mathrm{Fe}$ 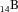
‐based magnets [11] in the 1980s, no new magnet material has exceeded its $BH_{max}$ performance. This discovery enabled compact hard drives that fueled the information age and lightweight motors that enabled drones and efficient electric transportation. Despite the expense and concerns associated with sourcing Nd, ${\mathrm{Nd}}_{2}\mathrm{Fe}$ 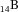 dominates roughly 60% of the magnet market, proving the importance of strong magnets. A new permanent magnet exceeding a $BH_{max}$ of 800 kJ/$m^{3}$, about twice that of ${\mathrm{Nd}}_{2}\mathrm{Fe}$ 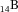, would revolutionize the industry, lead to ultra‐powerful motors and generators, and enable new, unforeseen applications (Figures 1 and 2).

The discovery of entirely new materials for even more powerful magnets is more likely now with the development of high‐throughput computational methods such as AI‐driven materials discovery and Density Functional Theory (DFT). A new magnet is likely to have an unknown, complex crystal structure with three or more distinct elements, like ${\mathrm{Nd}}_{2}\mathrm{Fe}$ 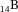. As the number of elements in a compound increases, the number of possible structures grows exponentially. While all of the elements and most binary (two‐element) compounds have been discovered and their magnetic properties characterized, ternary (three‐element) compounds are still being discovered, and most of the billions of four‐ and five‐element compounds are surely unknown [13]. With generative AI, millions of new compounds [14, 15] have been predicted to be stable, and DFT can estimate the essential magnetic performance before a material is even synthesized. The space of new materials is so large, however, that given the inherent uncertainties and experimental limitations an effective search must be focused, requiring some insight into the physics, chemistry and materials science of permanent magnets.

In this review, we will focus on the broad domain knowledge established over the last hundred years in the physics, chemistry, and materials science of magnetic materials, with the goal of formulating strategies to focus computational, high‐throughput searches. The computational methods themselves are comprehensively reviewed by Zhang [16]. Not only is it essential to incorporate domain expertise into a search, but the limits of domain knowledge can also be extended by computation. Thus, we also suggest where emergent phenomena and new magnet design principles could be uncovered by true machine learning.

## 2 Discussion

The physics of magnetic materials helps define what is possible, while new computational tools can now predict key performance metrics for a proposed material structure before the material is even synthesized.

The most successful conceptual framework for ferromagnets treats magnetic moments as localized on atoms due to unpaired electron spins. One unpaired spin per atom in a solid results in approximately 1 T of saturation magnetization, $B_{s}$ (Box 2). Good Fe‐based ferromagnets typically have one to two unpaired electrons per atom and therefore exhibit $B_{s}$ values of 1–2 T. High‐spin ${\mathrm{Mn}}^{2+}$ and ${\mathrm{Fe}}^{3+}$ ions can have five unpaired electrons per atom, while ${\mathrm{Gd}}^{3+}$ can have seven, so higher magnetization should, in principle, be possible. A new soft ferromagnet with $B_{s}>2.5$ T would find many specialty applications in high‐performance motors, transformers, and inductors for power conversion and electronics. Even if not particularly soft or hard, a new material with $B_{s}>2.5$ T might find application in creative motor designs that combine hard and soft magnet types, due to the higher magnetic field produced.

The magnetic atoms must be coupled in order for their moments to align in the same direction and form a ferromagnet (Box 2). The physical interaction that couples magnetic atoms is called the exchange interaction and determines whether spins align parallel (as in an ideal ferromagnet), antiparallel (as in an antiferromagnet), or adopt a more complex magnetic arrangement. Common ferrite ferromagnets actually have magnetic moments that align antiferromagnetically with neighboring atoms, but with different magnitudes so that they do not completely cancel; this is known as ferrimagnetism. For example, although the ${\mathrm{Fe}}^{3+}$ ions in ${\mathrm{Fe}}_{3}O$ 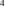 ferrite have five unpaired spins (5 $\mu_{B}$), the moments of neighboring ${\mathrm{Fe}}^{3+}$ and ${\mathrm{Fe}}^{2+}$ ions point in opposite directions. Combined with the large volume fraction (approximately half) of nonmagnetic oxygen, this limits the saturation magnetization of ferrites to at most about 0.5 $\mu_{B}$/atom, or roughly 0.5 T. Thus, while both ferromagnetic and antiferromagnetic exchange can produce a net ferromagnetic moment, ferromagnetic coupling is much more likely to yield a high‐$B_{s}$ magnet. In either case, for the magnetic ordering temperature (Curie temperature; Box 2) to be high enough for practical applications, the exchange interaction must be strong.

**FIGURE 3:**
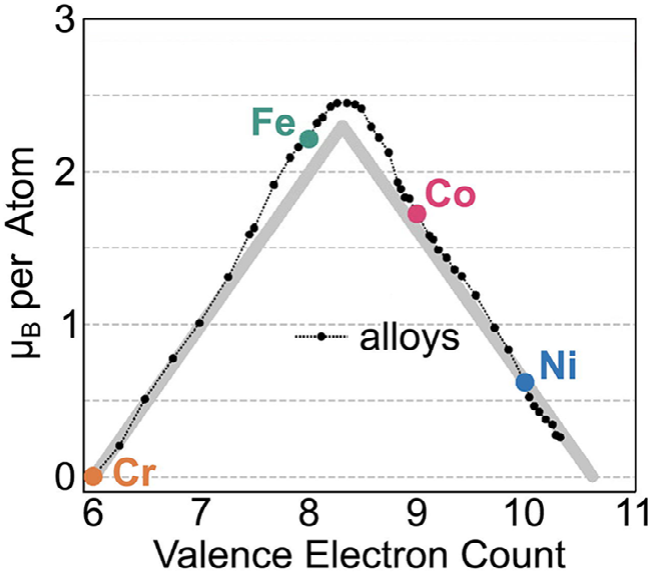
The saturation magnetization of 3d transition‐metal alloys peaks as a function of the number of electrons per atom. Adapted under the terms of the CC‐BY license [7]. Copyright 2024, Springer Nature.

Higher magnetization, up to about 2 $\mu_{B}$/atom or 2 T, is found in ferromagnetic metals such as alloys of Fe with Co or Ni. In metals, some electrons are delocalized, meaning their quantum states extend over many atomic sites, giving rise to high mobility and electrical conductivity. Delocalized electrons are shared by many atoms, are described by electron bands, and can also possess unpaired spins that contribute to magnetism. One key implication of the delocalized‐electron model is that strongly ferromagnetic electron bands often exhibit characteristics of localized electrons, such as relatively flat bands. As a result, magnetic metals such as Fe are often effectively described by localized magnetic moments with a non‐integer number of unpaired spins per atom [17]. The delocalized electrons mediate ferro‐ or antiferromagnetic alignment of neighboring spins through a mechanism known as indirect exchange. This model qualitatively explains trends such as why alloying Fe, Co, and Ni with other elements largely follows the average electron count per atom, with peak magnetization occurring between Fe and Co (Figure 3). In general, different metallic ferromagnets are expected to have an optimal electron concentration, which can be calculated using DFT. ${\mathrm{CeCo}}_{3}$, for example, undergoes a transition from nonmagnetic to ferromagnetic behavior [18] upon Mg doping, as explained by DFT calculations [19].

Although the local‐moment model has been quite useful in describing and predicting trends in ferromagnets for decades, it is the delocalized framework of Density Functional Theory (DFT), somewhat ironically, that has enabled major advances in predicting the magnetic properties of specific materials. Modern DFT methods have become extremely accurate for calculating the stability and ground‐state electronic structure of compounds, even when f‐electrons are involved [16, 20, 21]. In particular, zero‐temperature saturation magnetization, which is directly determined by the ground‐state spin polarization and electron count, is generally well captured by DFT across a wide range of magnetic materials. By contrast, calculating exchange interactions and, consequently, the temperature dependence of the magnetization below the Curie temperature–including an accurate determination of the Curie temperature itself–remains challenging and is not yet a standard or reliable outcome of conventional DFT approaches.

DFT calculations have progressed to the point that even a reasonable estimate of the maximum magnetic energy product ($BH_{max}$) is possible, such that much of the initial search for new magnetic materials can be conducted computationally. The energy barrier associated with changing the magnetization direction in a crystal is described by the magnetocrystalline anisotropy energy (MAE). Although these energy differences are small, they can be calculated with reasonable accuracy using DFT, particularly for materials without f‐electron elements. In equilibrium, the magnetization aligns along an easy crystallographic direction; reversal requires that the local magnetization pass through higher‐energy, hard directions under the influence of an applied magnetic field H. The field required to uniformly rotate the magnetization is the anisotropy field $H_{a}$, which is directly related to the MAE. In commercial permanent magnets, the coercivity $H_{c}$ typically reaches only about $H_{a}/4$ (Brown's paradox [5]) due to non‐uniform and defect‐mediated reversal mechanisms. Nevertheless, the anisotropy field–an intrinsic quantity accessible to DFT–provides a useful upper bound and a rough estimate of the coercivity and thus the attainable $BH_{max}$ for a proposed magnetic structure.

### 2.1 Strategies for New Magnets

While DFT can now accurately predict whether a given structure should be stable and whether it might be a good magnet, it does not provide precise guidance for finding the best magnet. With so many possible materials to choose from, structural and chemical trends are needed to narrow the search. The emphasis of this section is on identifying mechanisms that can produce unusually large saturation magnetization, a necessary condition for achieving magnetic energy densities above current benchmarks.

For example, certain elements–such as the 3d transition metals Cr, Mn, Fe, Co, and Ni, as well as rare earth elements with f‐electrons–are usually expected to exhibit some form of magnetism. From the many examples of antiferromagnetic and ferrimagnetic ionic insulators, it is often possible to estimate the magnetic moment of individual atoms. Typically, only transition metals with d‐electrons and rare earth elements with f‐electrons have unpaired spins, while s and p electrons usually pair with no net spin and therefore do not contribute to magnetism. Nonmetals such as oxygen are not magnetic because their s and p electrons are paired in filled shells or covalent bonding states and they possess no unpaired d or f electrons. The chemical trends and “rules” discussed here have many exceptions, suggesting that they could be expanded upon and rationalized through additional examples calculated by DFT and further categorized using machine learning comprehensive databases [22] with chemical and bonding descriptors.

**FIGURE 4:**
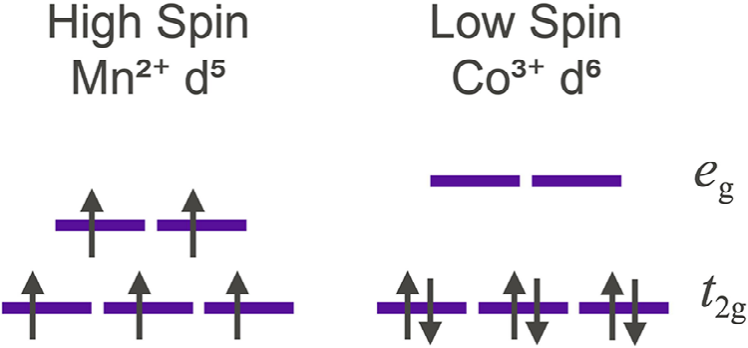
${\mathrm{Mn}}^{2+}$ often has five unpaired spins (left) due to strong Hund's rule exchange energy, resulting in a high saturation moment due to five unpaired spins per atom. In contrast, low‐spin ${\mathrm{Co}}^{3+}$ with no unpaired spins (right) has no magnetic moment because the crystal‐field splitting of the d‐electron states is large compared to the exchange energy.

The spin state of a transition metal ion typically depends on the number of d electrons and on the degree to which the d states are split in energy. Some ions, such as ${\mathrm{Mn}}^{2+}$, are usually high spin (Figure 4), with all five d electrons spin‐aligned as if the d orbitals were nearly degenerate, following Hund's rule. By contrast, octahedral ${\mathrm{Co}}^{3+}$ is typically low spin: all six d electrons are spin paired because the large crystal‐field splitting produces three low‐energy $t_{2g}$ orbitals that fill completely, making ${\mathrm{Co}}^{3+}$ usually nonmagnetic. The spin state trends with the type of ion, its charge (a higher oxidation state, e.g. ${\mathrm{Fe}}^{3+}$ vs ${\mathrm{Fe}}^{2+}$, is more likely to be low spin), and the strength of bonding in the local environment, often characterized by a spectrochemical series of ligands.

Some nonmagnetic or weakly magnetic elements might also contribute to an ultra‐strong magnet. The 4d and 5d transition metals are usually low spin or form nonmagnetic metals; however, these heavier elements exhibit stronger spin–orbit coupling, which can enhance magnetocrystalline anisotropy (MAE). This makes heavy alkaline earth elements such as Sr and Ba, earlier transition elements such as Zr, Hf, Nb, Ta, and W, or even very heavy elements such as Bi, Pb, Th, or U potentially attractive components. Finally, including small interstitial elements with low atomic volume, (e.g., producing suboxidized structures discussed below) may promote superexchange interactions and stabilize distinct structure types.

The magnetism of rare earth elements differs sufficiently from that of transition metals that a separate set of guidelines is useful. The weakly interacting f‐shell electrons in rare earth elements are almost always high spin, adopting the maximum spin configuration. However, this weak interaction also permits a substantial orbital angular momentum contribution to the atomic magnetic moment. The lighter rare earth elements (fewer than seven f electrons) typically have opposing spin and orbital contributions, while the heavier rare earth elements (more than seven f electrons) have spin and orbital contributions aligned. As a result, ${\mathrm{Gd}}^{3+}$, with seven unpaired electrons and no orbital contribution, has a magnetic moment of 7 $\mu_{B}$, whereas heavy rare earths such as ${\mathrm{Dy}}^{3+}$ and ${\mathrm{Ho}}^{3+}$ can reach moments of up to ∼10 $\mu_{B}$/atom due to the orbital contribution. However, in many materials such as $R_{2}\mathrm{Fe}$ 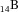 (R = rare earth elements such as Nd, Gd, or Dy), the light rare earth elements align ferromagnetically at room temperature and enhance the magnetization, while the heavy rare earth elements align antiferromagnetically and reduce it. The spin‐orbit interaction of the rare earth atoms is often associated with the strong magnetocrystalline anisotropy needed to make a hard magnet. By contrast, the d electrons in transition metals typically have little or no orbital contribution to magnetism due to strong hybridization with neighboring atoms.

The exchange coupling in ionic materials, however, is often, but not always, antiferromagnetic. Thus, even when the magnetic moment on each atom is large, neighboring moments often cancel, yielding a small net magnetization. In ionic materials, the exchange interaction between magnetic atoms is usually mediated through a nonmagnetic anion (e.g., $O^{2-}$ in oxides) via superexchange, which occurs through metal–nonmetal bonds.

**FIGURE 5:**
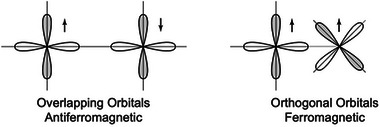
Goodenough‐Kanamori rule predicting strong antiferromagnetic coupling for orbitals with unpaired spins that directly overlap (as if making bonding and antibonding orbital with paired spin bonding orbital as ground state), and weak ferromagnetic coupling when the orbitals are orthogonal (like orbitals that do not bond and instead form two degenerate states with parallel spins by Hund's rule). Adapted with permission [25]. Copyright 2014, Cambridge University Press.

The Goodenough–Kanamori rules [23, 24] provide qualitative guidance for whether interactions are ferromagnetic or antiferromagnetic based on atomic structure and bond angle [25]. For example, direct coupling between overlapping orbitals with unpaired spins (e.g., two ${\mathrm{Mn}}^{2+}$ atoms) is expected to be strongly antiferromagnetic (Figure 5), each spin state lowering its energy by having a greater spatial extent, like a spin‐paired covalent bond. If the orbitals hosting unpaired spins are orthogonal (non‐overlapping), the coupling can be ferromagnetic like two orthogonal, degenerate states with parallel spins due to Hund's rules. Such ferromagnetic coupling may occur when two different magnetic atoms host unpaired spins in orthogonal orbitals (e.g., Cr with unpaired spins in $t_{2g}$ orbitals and Co with unpaired spins in $e_{g}$ orbitals). This suggests a strategy of alternating magnetic species, such as layered Pt and Co in PtCo or Mn and Al in L1
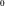
‐structured MnAl [26].

**FIGURE 6:**
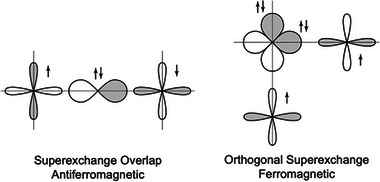
Goodenough‐Kanamori rule for superexchange predicting strong antiferromagnetic coupling when unpaired spins interact through mutually overlapping intermediary orbitals (e.g., an oxygen p orbital in a 180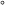 Fe–O–Fe bond), but weak ferromagnetic coupling when the interacting orbitals are orthogonal (e.g., different, orthogonal p‐orbitals interact with Fe in a 90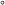 Fe–O–Fe bond angle. Adapted with permission [25]. Copyright 2014, Cambridge University Press.

A nonmagnetic atom between magnetic atoms, for example oxygen in iron oxides, can mediate a similar interaction via superexchange (Figure 6). A 180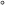 Fe–O–Fe bond angle leads to strongly antiferromagnetic coupling, whereas a 90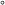 bond angle favors ferromagnetic coupling because the oxygen p orbital overlapping with one Fe atom is orthogonal to the other Fe atom.

Although metals often make good magnets, robust design rules for metallic ferromagnets are still lacking. From the elemental magnetic metals (Fe, Co, Ni) and their alloys, it is clear that indirect exchange mediated by metallic conduction electrons can be strong and can lead to ferromagnets with high Curie temperatures. Although metallic iron, ${\mathrm{Fe}}^{0}$, is effectively in a spin state with only two unpaired spins per atom [17], its saturation magnetization is relatively high, ∼2 T, because there are no nonmagnetic atoms diluting the magnetic sublattice. Indirect exchange is also known to be highly sensitive to crystal structure: body‐centered cubic (BCC) α‐iron (pure iron at room temperature) is ferromagnetic, whereas face‐centered cubic (FCC) γ‐iron (found, for example, in many stainless steels) is not [27]. The Ruderman–Kittel–Kasuya–Yosida (RKKY) mechanism of indirect exchange is known to oscillate between ferromagnetic and antiferromagnetic coupling as a function of interatomic distance. Because DFT can accurately predict magnetic ground states, useful design principles may emerge from large‐scale computational searches combined with machine‐assisted learning. For example, antiferromagnetic interactions might be geometrically frustrated by specific lattice motifs (such as Kagome lattices) while maintaining sufficient net ferromagnetic exchange to produce a strong magnet.

**FIGURE 7:**
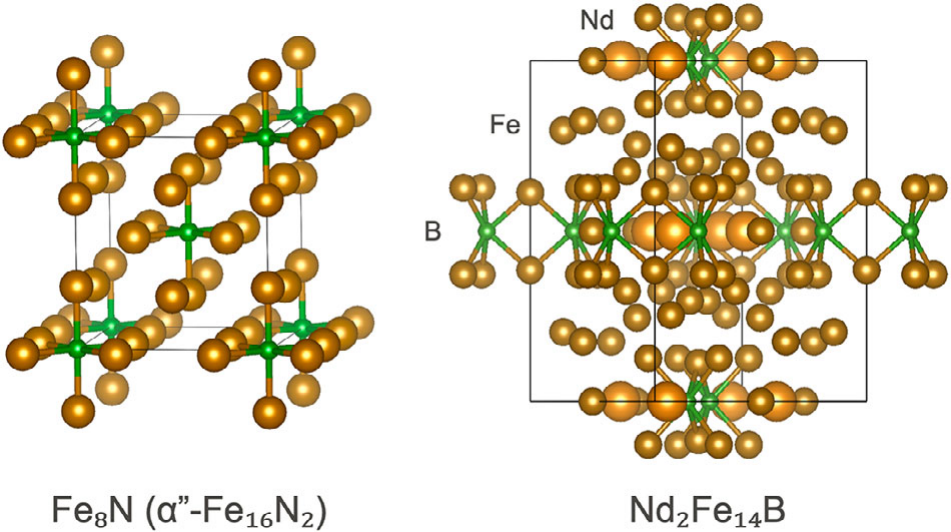
Crystal structures of ${\mathrm{Fe}}_{8}N$ ($\alpha^{\prime \prime }$‐${\mathrm{Fe}}_{16}N$ 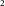) and ${\mathrm{Nd}}_{2}\mathrm{Fe}$ 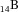, highlighting the ${\mathrm{Fe}}_{6}N$ subnitride octahedra and ${\mathrm{Fe}}_{6}B$ subboride trigonal prisms.

The strongest commercial permanent magnet today, ${\mathrm{Nd}}_{2}\mathrm{Fe}$ 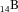, combines both ionic bonding and metallic character within a complex, anisotropic crystal structure–features that may be essential to its exceptional performance (Figure 7). ${\mathrm{Nd}}_{2}\mathrm{Fe}$ 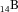 is approximately 82% iron, so it is not surprising that its saturation magnetization is comparable to that of pure α‐iron. However, α‐iron and its alloys exhibit low magnetocrystalline anisotropy, as expected for cubic materials, which leads to low coercivity $H_{c}$ and modest $BH_{max}$. By contrast, ${\mathrm{Nd}}_{2}\mathrm{Fe}$ 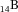 has a distinctly layered structure, with planes containing Nd and B separating thick Fe‐rich layers. While the large magnetocrystalline anisotropy is often attributed to the strong spin–orbit interaction of the rare earth element (e.g., Nd), the $R_{2}\mathrm{Fe}$ 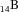 structure also forms with nominally nonmagnetic rare earths such as Y, La, Ce, Lu, and Th, yet still exhibits magnetocrystalline anisotropy an order of magnitude larger than that of metallic Fe [11]. This suggests that the inherently anisotropic crystal structure itself plays a dominant role in establishing large MAE.

**FIGURE 8:**
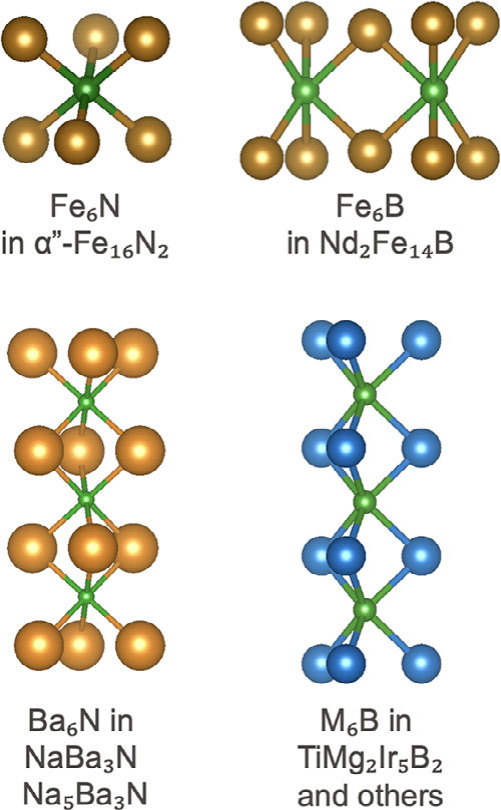
Chains of subnitrides are also known to exist and would likely exhibit high magnetocrystalline anisotropy if magnetic. ${\mathrm{NaBa}}_{3}N$ [28] and ${\mathrm{Na}}_{5}\mathrm{Ba}$ 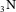 [29] contain chains of ${\mathrm{Ba}}_{6}N$ octahedra similar to the ${\mathrm{Fe}}_{6}N$ subnitride octahedra in ${\mathrm{Fe}}_{8}N$ ($\alpha^{\prime \prime }$‐${\mathrm{Fe}}_{16}N$ 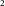). Many borides such as ${\mathrm{TiMg}}_{2}\mathrm{Ir}$ 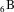 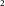 [30] contain chains of $M_{6}B$ trigonal prisms similar to the ${\mathrm{Fe}}_{6}B$ subboride trigonal prisms in ${\mathrm{Nd}}_{2}\mathrm{Fe}$ 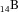.

The polar‐covalent Fe–B bonds in ${\mathrm{Nd}}_{2}\mathrm{Fe}$ 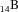 may not only help enforce its anisotropic crystal structure, but also give rise to essential magnetic interactions. The boron atoms in ${\mathrm{Nd}}_{2}\mathrm{Fe}$ 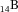 form short (∼2.1 Å) bonds to six neighboring Fe atoms. This trigonal prismatic ${\mathrm{Fe}}_{6}B$ structural unit (Figure 8) strongly distorts the surrounding close‐packed iron layers, which should influence indirect exchange interactions and contribute to uniaxial anisotropy. The trigonal prismatic ${\mathrm{Fe}}_{6}B$ unit is closely related to the octahedral ${\mathrm{Fe}}_{6}N$ unit found in iron subnitrides and can be described as a subboride motif.

The suboxidized (not fully oxidized, yet still metallic) forms of magnetic elements may combine beneficial aspects of ionic (or polar‐covalent) and metallic bonding in ferromagnets. Iron subnitride ${\mathrm{Fe}}_{8}N$ (commonly referred to as $\alpha^{\prime \prime }$‐${\mathrm{Fe}}_{16}N$ 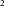), for example, contains an $N^{3-}$ ion surrounded by six Fe atoms in an ${\mathrm{Fe}}_{6}N$ octahedron (Figures 7 and 8), which can facilitate superexchange interactions similar to those in oxides. The $N^{3-}$ anion removes three electrons from the otherwise metallic system, providing an additional mechanism to tune the average electron count per Fe atom for indirect exchange. However, $\alpha^{\prime \prime }$‐${\mathrm{Fe}}_{16}N$ 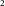 lacks an obviously layered structure and therefore, unsurprisingly, exhibits much lower magnetocrystalline anisotropy than ${\mathrm{Nd}}_{2}\mathrm{Fe}$ 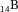.

Known subnitrides and suboxides [31] exhibit a wide range of arrangements of suboxidized octahedra, suggesting that many additional anisotropic structures may be possible. The suboxides ${\mathrm{Rb}}_{9}O$ 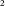 and ${\mathrm{Cs}}_{11}O$ 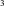 contain clusters of two or three face‐sharing $M_{6}O$ octahedra (Figure 8), which are significantly less isotropic than isolated octahedra. ${\mathrm{NaBa}}_{3}N$ [28] and ${\mathrm{Na}}_{5}\mathrm{Ba}$ 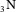 [29] form linear chains of face‐sharing ${\mathrm{Ba}}_{6}N$ octahedra, which would be expected to produce very large anisotropy if realized with ${\mathrm{Fe}}_{6}N$ units. Indeed, linear 3d Fe‐based compounds have been identified with extraordinary magnetocrystalline anisotropy and large magnetic moments per Fe atom [32].

Similar suboxidized motifs exist with other anions such as B and C and can therefore be described as subborides and subcarbides. Other small, electronegative elements such as Be, Si, and P can also form related structural units. Beyond ${\mathrm{Nd}}_{2}\mathrm{Fe}$ 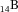, many other magnetic borides with a wide variety of structural motifs are known and continue to be discovered [30]. Several borides contain 1D chains of $M_{6}B$ trigonal prisms (Figure 8) and exhibit large magnetocrystalline anisotropy. The related carbide ${\mathrm{Nd}}_{2}\mathrm{Fe}$ 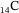 also forms, along with many iron carbides in which carbon occupies the center of ${\mathrm{Fe}}_{6}C$ octahedra, suggesting the potential for complex subcarbide chemistry. Incorporating additional magnetic elements into this rich set of suboxidized structural motifs defines a very large, yet still chemically focused, space of unexplored materials. The coexistence of ionic, polar‐covalent, and metallic bonding can produce diverse exchange interactions, while the resulting complex structures naturally introduce anisotropy essential for the large MAE required in hard magnets.

Several new magnetic materials with three or more elements and complex crystal structures have been discovered in recent years, indicating that this materials space remains far from fully explored. Examples include, in addition to borides [30], newly reported nitrides [33], carbides [34], phosphides [35], and intermetallic compounds [36]. Such complex materials often do not melt congruently and therefore require solid‐state annealing or other low‐temperature processing routes, much like ${\mathrm{Nd}}_{2}\mathrm{Fe}$ 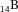. Notably, some of these compounds were first identified computationally prior to experimental realization [34].

A systematic search typically entails mapping thermodynamically stable phases within an equilibrium phase diagram. Synthesizable materials are generally stable at some growth condition (temperature, composition) [37], such that a search guided by equilibrium phase diagrams could, in principle, discover all possible compounds, analogous to finding new countries on a map. Although the serendipitous discovery of ${\mathrm{Nd}}_{2}\mathrm{Fe}$ 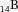 in the 1980s did not result from a systematic exploration of chemistry or ternary phase space, it nevertheless prompted extensive subsequent searches, particularly among rare‐earth–transition‐metal binary phases and their derivatives [38]. There are expected to be thousands of relevant ternary and quaternary systems of magnetic elements [39], encompassing millions of possible compounds [13], that remain largely unexplored.

Until recently, a systematic search required synthesizing and characterizing large numbers of samples with varying compositions processed at different temperatures. Experimentally, new thermodynamic phases are identified simply by combining elements, equilibrating them at a given temperature, and detecting the phases that segregate. In principle, this process could be simulated using molecular dynamics; however, tracking atomic motion over the time scales required for nucleation and growth of competing phases is computationally prohibitive.

Consequently, most compounds discovered computationally begin from known crystal structures in which atoms are substituted with chemically similar elements, mirroring common strategies in experimental solid‐state chemistry. When such systems have not already been thoroughly explored experimentally (e.g., binaries or well‐studied ternary magnets), this approach can be efficient and relatively reliable [16]. The phase space of possible substitutions becomes very large when many elements are involved, as in high‐entropy alloys. However, the dominant effects often reduce to well‐understood mechanisms such as local‐moment substitution (e.g., ferrites and garnets), valence‐count adjustment (Fermi‐level tuning in metals), and chemical pressure effects arising from changes in lattice parameters. Relatively small compositional changes can nevertheless lead to dramatic effects, such as the emergence of ferromagnetism in ${\mathrm{CeCo}}_{3}$ or improved phase stability in MnAl through carbon addition [40].

The principal computational challenge remains the discovery of entirely new crystal structures; however, new computational approaches are developing rapidly. The use of adaptive genetic algorithms has already predicted several new magnetic compounds that were subsequently confirmed experimentally [34]. With the rapid development of artificial intelligence [14, 15], the discovery of new crystal structures is poised to accelerate further, particularly as automated laboratories are developed for experimental verification [41, 42].

What remains uncommon computationally is the chemical strategy of discovering new structures by utilizing known structural motifs, such as ${\mathrm{Fe}}_{6}B$ subboride units connected in novel ways. With the ability of AI to recognize structural patterns and combine them in new ways, it may become possible to rapidly conceive new structures and then test their stability and magnetic performance using density functional theory without extensive experimental screening. By calculating the forces on each atom, the system can be allowed to relax into a locally stable structure. The magnetic ground state (e.g., ferromagnetic versus antiferromagnetic) is then determined by comparing the energies of different magnetic configurations. Determining global thermodynamic stability additionally requires evaluating competing structures of differing composition. Even if only locally stable, identifying new ultra‐strong magnetic structures may reveal promising chemical and structural strategies for ultimately realizing a globally stable ultra‐strong magnet.

To efficiently identify new three‐element, four‐element, and higher‐complexity structures, directed high‐throughput methodologies should be employed. The use of probable chemistries and structural motifs should help limit the search space. If a search methodology would not have been capable of identifying ${\mathrm{Nd}}_{2}\mathrm{Fe}$ 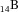 (e.g., due to its large unit cell), it is unlikely to be sufficient. The saturation magnetization (at least at 0K) can be readily calculated by DFT for ferromagnetic configurations, but antiferromagnetic and ferrimagnetic alternatives must also be evaluated to identify the true magnetic ground state. Assessing stability relative to competing phases is equivalent to determining the relevant equilibrium phase diagrams. This suggests that a successful effort should focus on a targeted chemical phase space coupled with rapid experimental verification.

**FIGURE 9:**
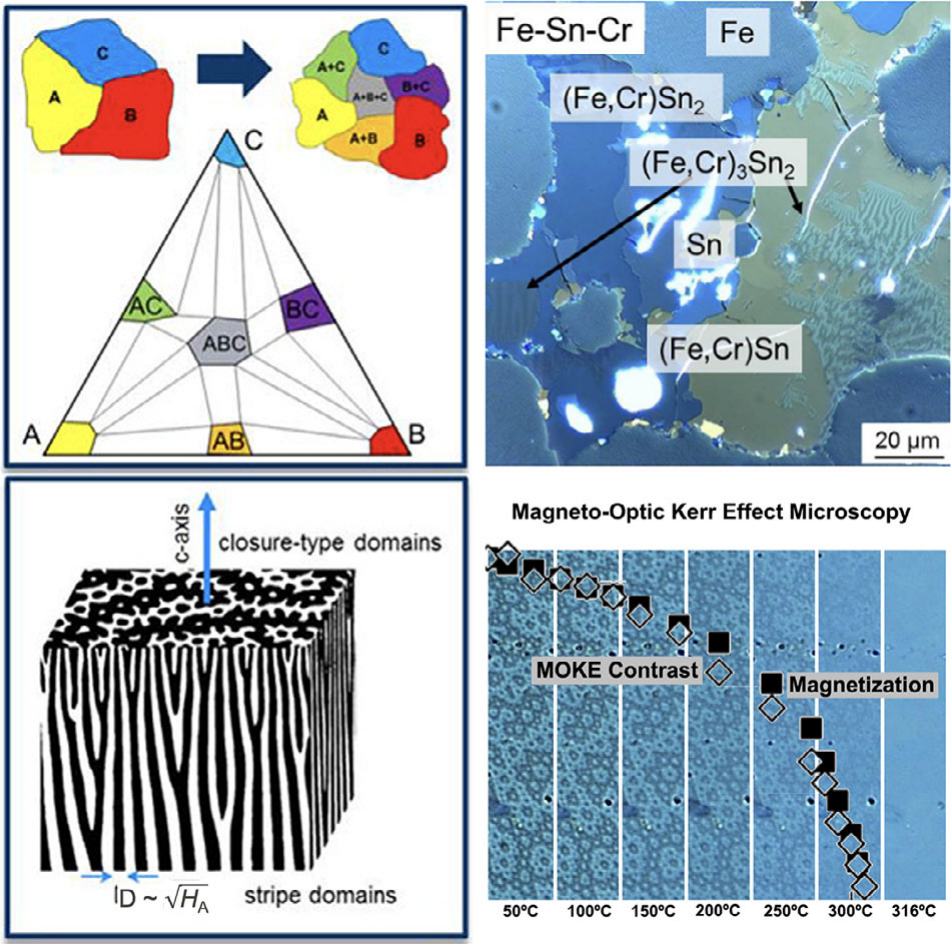
Diffusion couples as a high‐throughput search strategy for new magnets. Incomplete reaction between elements A, B, and C can lead to the formation of all possible phases–binary (AB, AC, BC) and ternary (ABC)–within the phase diagram (a). Using polarized light. Magneto‐optic Kerr Effect microscopy (MOKE), the different phases can be identified, including the magnetic domain structure in ferromagnetic phases such as (Fe,Cr)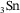 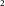 (b). The anisotropy field $H_{A}$ can be estimated from the magnetic domain spacing D (c). The Curie temperature $T_{C}$ can be estimated from the decrease in MOKE‐intensity domain contrast with increasing temperature (d). Adapted with permission [44]. Copyright 2018, Elsevier [39]. Copyright 2014, IOP Publishing.

High throughput experiments might also help accelerate the discovery process. In principle, multiple diffusion couples within a single sample can be used (Figure 9) to characterize an entire phase diagram [43], with magnetic properties probed using scanning microscopy techniques [44]. Thin‐film techniques also exist; however, phase stability and magnetic properties in thin films do not always translate directly to bulk materials, and most applications ultimately require ${\mathrm{cm}}^{3}$‐scale samples. Once a new, promising phase is discovered, traditional synthesis and characterization methods should be sufficient to quickly identify a processing route to a commercial magnet. Within a year after ${\mathrm{Nd}}_{2}\mathrm{Fe}$ 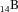 was identified as a good magnet material, several companies were preparing commercial magnets [45].

Focusing on new, thermodynamically stable structures with high saturation magnetization ($B_{s}>2$–2.5 T) is likely the most important initial step toward discovering an ultra‐powerful magnet. For a hard magnet to achieve $BH_{max}$ > 800 kJ/$m^{3}$, the saturation magnetization $B_{s}$ must be at least 2 T because $BH_{max}$ is fundamentally limited by $B_{s}$ ($BH_{max}<B_{s}^{2}/4\mu_{0}$), regardless of how large the anisotropy field $H_{a}$ may be [46]. Although a Curie temperature above 200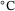 is required for most applications, it may not be a critical initial criterion, as a material with a calculated $B_{s}>2.5$ T is likely to exhibit strong exchange coupling and is therefore worth synthesizing and measuring. While it should be possible to calculate the anisotropy field $H_{a}$ (from the MAE) as a proxy for the maximum achievable coercivity, an anisotropic crystal structure with anisotropic bonding may be a sufficient indicator of high MAE. Experimental verification of $B_{s}$ values exceeding 2–2.5 T would likely generate sufficient momentum for rapid processing development, advancing the project into a new phase.

### 2.2 Processing Magnets

All commercial magnet production processes are optimized to control the material microstructure, whether the goal is a hard or a soft magnet, because microstructure strongly affects the coercivity $H_{c}$. High coercivity and $BH_{max}$ in hard magnets usually require small grains that are preferentially aligned in a magnetic field. Large single crystals have low coercivity because opposing magnetic domains can easily nucleate and grow by domain wall motion in an opposing magnetic field. Small, single‐domain crystals, typically 50 nm to 5 μm in size, are usually most effective at hindering the nucleation and growth of opposing magnetic domains. Embedding such particles in a polymer or elastomer to make a bonded magnet is relatively simple, but the added nonmagnetic volume reduces the saturation magnetization $B_{s}$. To achieve high $B_{s}$, the particles should also be crystallographically aligned by applying a magnetic field while the polymer is still sufficiently fluid to allow particle rotation. The highest‐$B_{s}$ magnets are sintered magnets, in which small particles are aligned, pressed, and sintered rapidly at high temperature to suppress grain growth. The resulting grain boundaries, typically only a few nanometers thick, have compositions and properties that differ from those of the bulk magnet. Optimizing processing conditions and composition, including the use of grain‐boundary diffusion additives, can greatly improve the coercivity. After cutting to the desired shape, magnets are often coated to provide environmental protection (e.g., against air or water exposure) and improved mechanical robustness.

**FIGURE 10:**
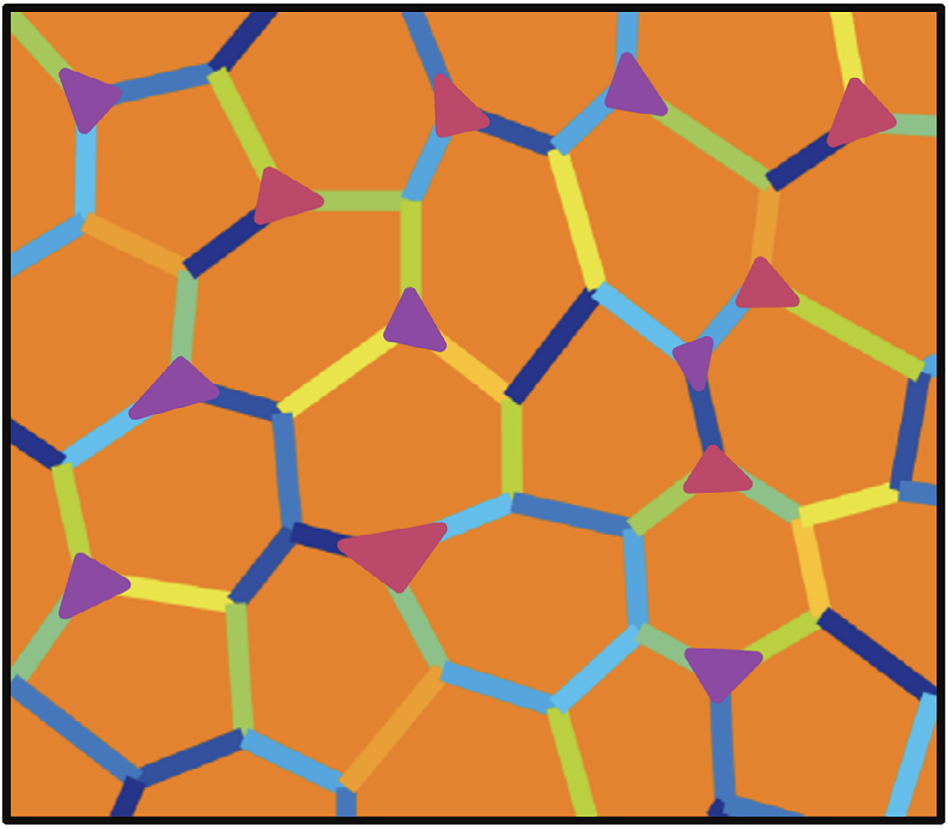
In thermodynamic equilibrium, the microstructure of a complex magnet will generally contain multiple phases. The strong magnetic phase (orange) should occupy the majority of the volume, but multiple bulk (3D) impurity phases (triangles) are usually also present, often at triple junctions that reflect the chemical potentials during processing. Thin (1–10 nm) 2D grain‐boundary phases (complexions) exist at grain boundaries (lines) and are essential for achieving high coercivity in a permanent magnet. Their structure and properties vary with grain‐boundary character and chemical potential, linking them to the more easily observed 3D impurity phases that also form between grains.

Even in thermodynamic equilibrium, the microstructure of a complex magnet material will contain both impurity phases and grain‐boundary phases (Figure 10). Because of the limited compositional range of stability and practical processing considerations, impurity phases are generally present, usually at grain boundaries and triple junctions. These impurities are bulk (3D) phases in equilibrium with the magnet material and are determined by the chemical potentials. Which impurity phases are present varies with chemical composition and can be identified using phase‐boundary mapping of equilibrium phase diagrams [47]. The equilibrium interface between two crystals is a 2D phase with a distinct composition, referred to as a grain‐boundary phase or a *complexion*, to distinguish it from the 3D impurity phases at grain boundaries, which are also sometimes called grain‐boundary phases [48]. It is generally believed that nonmagnetic complexions are essential to forming a hard, sintered magnet such as ${\mathrm{Nd}}_{2}\mathrm{Fe}$ 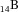 [49, 50]. The 3D impurity phases (and voids) at the grain boundaries, however, are not beneficial, because they directly reduce $B_{s}$ by displacing magnetically active material. This microstructure should remain stable in the absence of grain growth.

A magnet microstructure can also contain non‐equilibrium features that may enhance macroscopic magnetic properties, motivating the development of grain‐boundary diffusion additives [51]. A composition gradient within a single crystal grain or across the macroscopic scale of a sample indicates that atomic diffusion was quenched before global equilibrium was reached. Nevertheless, locally the 2D and 3D phases present may still be well described by an effective equilibrium temperature and chemical potential.

## 3 BOX 1 ‐ Magnets in Motors and Generators

**FIGURE 11:**
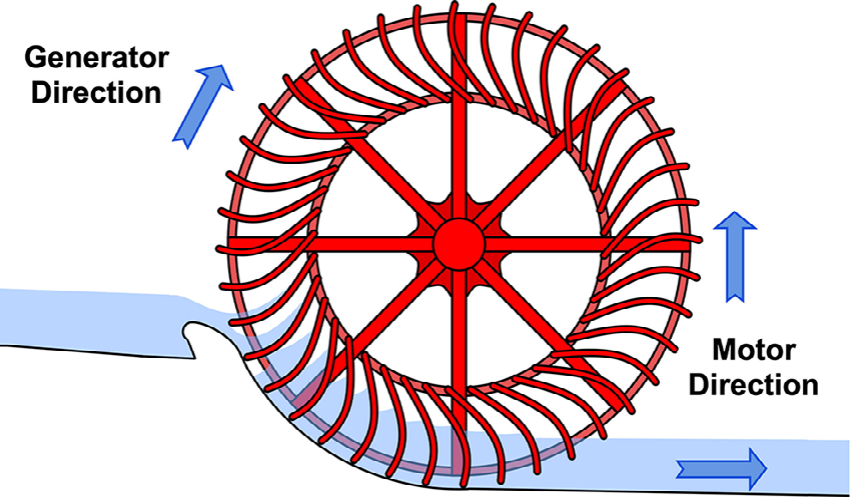
In an electric generator, magnets push electrons to create an electric potential (voltage), analogous to a paddle wheel lifting water to a higher gravitational potential. In a motor, flowing electrons push a rotating wheel of magnets, analogous to water flowing downstream driving a water wheel. Adapted under the terms of the CC0‐1.0 license [52].

The function of a magnet in a motor or generator is analogous to a paddle‐wheel pushing water (Figure 11). As a magnet is moved close to a coil of wires, it pushes the electrons in the wires, like a paddle can push water upstream. Just like a moving paddle‐wheel can lift water to a higher gravitational potential, the motion of a magnet in a generator produces an electric potential [electro‐motive force (EMF)] measured as a voltage. A larger magnetic field B is like a larger paddle (force or torque in a motor is proportional to B). The mechanical energy of the paddle is reduced to lift the water increasing the water's gravitational energy. In the same way, the mechanical energy of a spinning wheel of magnets in a generator is converted to electric potential energy.

Conversely, like moving or falling water can push a paddle wheel to convert gravitational energy of the water to mechanical energy of a water wheel, electrons in a coil produce a magnetic field that push a magnet turning the wheel of a motor. Thus we can convert the mechanical energy of a combustion engine to electricity at one location producing electric voltage that we then conduct long distances along wires to power a motor converting electric energy back into mechanical energy.

When the paddle pushes water, the water pushes back due to Newton's law of opposing forces. The magnet must be strong enough (mechanically and magnetically) to withstand the opposing force in the same way a paddle needs to be strong enough to push water without bending. Thus a magnet producing a strong magnetic field (B) must be able to withstand a similar magnitude reverse, applied field (H). A strong permanent (hard) magnet then has not only a large saturation moment $B_{s}$ but also a large Coercive field $H_{c}$ = the applied field required to flip the magnet.

In Energy applications magnets are often compared to batteries: Whereas a battery stores electrical energy and then becomes depleted, permanent magnets help interconvert mechanical and electrical energy without being drained by repeated use [1].

## 4 BOX 2 ‐ Hysteresis Loops and Curie Temperature

Ferromagnets have a spontaneous magnetic moment due primarily to unpaired electron spins at the atomic scale [1]. 1 This net magnetic moment is a vector that prefers to align along a particular crystallographic direction, known as the easy direction, but can be forced into another direction by an external magnetic field, H. Magnetic domains are regions within a single crystalline grain in which the net magnetic moments are aligned in the same direction. The maximum internal magnetic field produced spontaneously (H=0) by a ferromagnet is the saturation magnetization, $B_{s}$.

**FIGURE 12:**
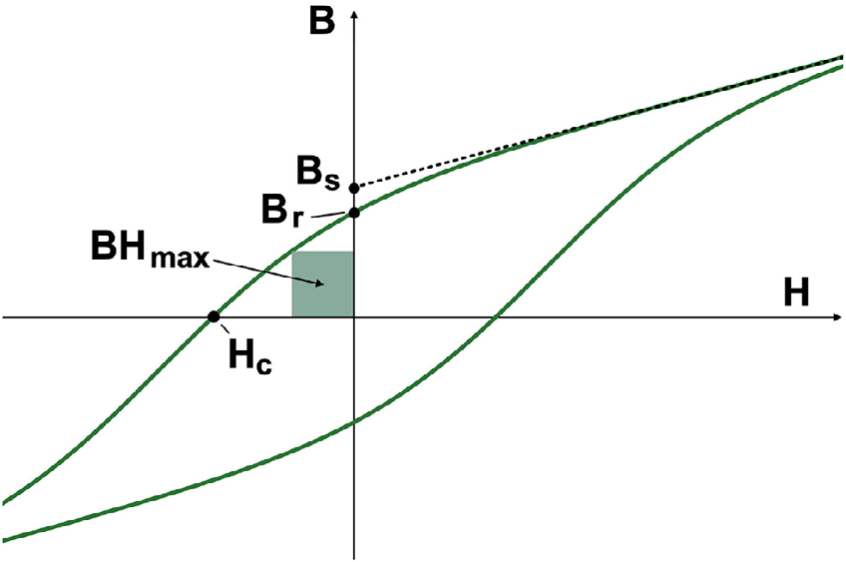
Hysteresis loop (B vs. H) of a hard ferromagnet showing saturation magnetization, $B_{s}$ (extrapolated to zero applied field), remanent magnetization, $B_{r}$, coercivity, $H_{C}$ (where B=0), and maximum energy product $BH_{\max}$.

The overall magnetization of a bulk magnet is the vector sum of all magnetic domains with varying magnetic and crystallographic orientations. The magnetic field B, is the sum of magnetization and applied field H. How the magnetization changes with applied magnetic field, and therefore how it can be used, is characterized by a hysteresis loop (Figure 12).

A sufficiently large applied field will force all magnetic domains to align along the field direction, allowing a measurement of the saturation magnetization $B_{s}$. Here we define $B_{s}$ as the linear extrapolation of B versus H to H=0 in the high‐field saturation regime. As H is reduced, the magnetization within each grain may rotate toward the preferred (easy) crystallographic direction or form reverse domains due to the opposing internal demagnetizing fields arising from neighboring regions. As some domains reorient or change size, the total magnetization decreases. When the applied field is removed (H=0), a finite magnetization, called the remanent magnetization, remains.

Reversing the applied field H causes additional domain wall motion favoring reverse oriented domains. Eventually, a sufficiently strong opposing field reduces the magnetic field (B=4πM+H) to zero; this applied field is called the coercive field $H_{c}$. Cycling the applied field from large positive to large negative values and back produces a B–H hysteresis loop (Figure 12). Magnetization reversal occurs primarily through motion of the boundaries between domains within a single crystalline grain, known as domain walls. Domain wall motion can be hindered by magnetocrystalline anisotropy, microstructural defects, and secondary phases, making $H_{c}$ strongly dependent on material processing.

Magnets with wide hysteresis loops and large $H_{c}$ are classified as hard magnets. Permanent magnets are hard ferromagnets that retain their magnetization after the alignment field is removed and during operation. In hard magnets, strong pinning of domain walls inhibits their motion. A common figure of merit for permanent magnets, which requires both large $B_{s}$ and $H_{c}$, is the maximum energy product $BH_{max}$, defined as the largest product of B and H in the quadrant of the hysteresis loop where B opposes H. Maintaining large torque in motors or generators requires a high magnetic induction B to persist in the presence of a reversed magnetic field H.

Soft magnets, in contrast, have narrow hysteresis loops due to facile domain wall motion. They are used in applications requiring rapid magnetic field switching, such as electrical transformers, where energy loss is directly related to the width of the hysteresis loop.

**FIGURE 13:**
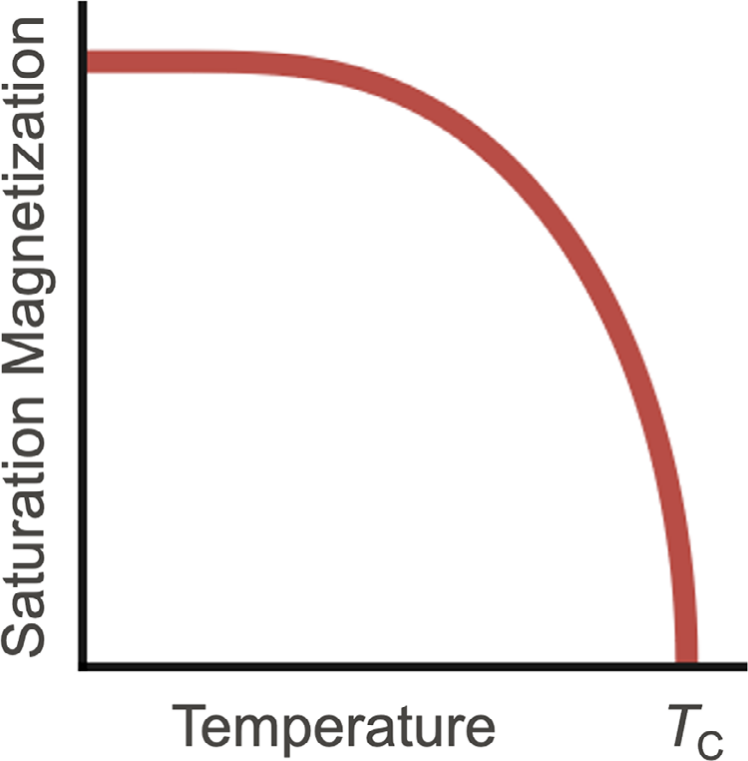
Saturation magnetization $B_{s}$ of a ferromagnet decreases with temperature, vanishing at the Curie temperature, $T_{C}$.

The spontaneous magnetic moment of a ferromagnet fluctuates due to thermal energy (Figure 13), leading to a reduction in the saturation magnetization $B_{s}$ as temperature increases. At a critical temperature, called the Curie temperature $T_{c}$, long‐range magnetic order is lost and the spontaneous moment vanishes. Above $T_{c}$, atomic spins randomly fluctuate rapidly, producing no net magnetization; the material undergoes a transition from a ferromagnetic to a paramagnetic state.

## 5 Conclusion

Considering the vast chemical design space available to magnetic materials and the intrinsic complexity of magnetism, the discovery of new, substantially stronger permanent magnets should not only be just possible, but likely. There is no simple recipe for optimizing magnetic performance, as multiple exchange mechanisms and competing interactions depend sensitively on crystal chemistry, coordination environments, and electron count. A new magnet, much like ${\mathrm{Nd}}_{2}\mathrm{Fe}$ 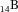, probably contains three or more distinctly different elements and/or structures, which we call a complex magnet material. Given the continued discovery of new structures even in the past decade, there are likely many still waiting to be discovered. In particular, the existence of atoms with five or more unpaired spins suggests that saturation magnetizations exceeding those of metallic iron alloys are physically achievable, with disruptive impact anticipated for materials surpassing saturation magnetization, $B_{S}$, greater than 2.5T or maximum energy product, $BH_{max}$, greater than 800kJ/$m^{3}$.

Thanks to recent advances in computational materials discovery [53, 54], much of the search for a new magnet can now be done computationally much faster. With Density Functional Theory (DFT) one can predict the formation energy of any proposed crystal structure with sufficient accuracy to predict thermodynamic stability and even the magnetic structure and saturation magnetization. Equilibrium phase diagrams can be used to provide a comprehensive map of phases allowing for a systematic study.

With so many possibilities, an effective search for a new magnet will undoubtedly focus using a physical and/or chemical strategy. While recent efforts have emphasized magnets composed solely of earth‐abundant elements, this constraint significantly narrows the accessible design space and may exclude the most powerful candidates. The rapid commercial success of ${\mathrm{Nd}}_{2}\mathrm{Fe}$ 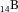 following its discovery illustrates the transformative value of a genuinely superior magnet that could unlock ultrapowerful motors, more efficient generators, and applications yet to be envisioned for a new generation of complex magnet materials.

## Conflicts of Interest

The authors declare no conflicts of interest.

## Data Availability

The author have nothing to report.

## Endnotes

1 In 3d transition metals, the orbital angular momentum is strongly reduced, “quenched,” by the crystal field, so that the saturation magnetization is dominated by the spin contribution. For rare‐earth magnets, the orbital angular momentum of the localized 4f electrons is only partially quenched and contributes to the magnetism.
